# Unusual clinical manifestation of seborrheic keratosis on the scalp successfully treated with topical trichloroacetic acid: an atypical case report

**DOI:** 10.11604/pamj.2020.37.98.26090

**Published:** 2020-09-28

**Authors:** Dewi Nur Komalasari, Khairuddin Djawad, Airin Riskianty Nurdin, Idrianti Idrus Paturusi

**Affiliations:** 1 Department of Dermatology and Venereology, Faculty of Medicine, Hasanuddin University, Hasanuddin University Hospital, Makassar, South Sulawesi, Indonesia

**Keywords:** Dermoscopy, histopathology, scalp seborrheic keratosis, trichloroacetic acid

## Abstract

Seborrheic keratoses (SKs) are benign tumor of the skin. They can occur anywhere, except the palms and soles. SKs commonly present as multiple, pigmented papules which is classically described as verrucous stuck-on papules or plaques. A case with multiple nodular appearance is a rare case and to our knowledge has never been reported. Here we report a rare case of multiple SKs on the scalp. On clinical evaluation, multiple, erythematous hyperkeratotic nodules were present. Dermoscopic and histopathological examination supported SKs. The patient was successfully treated with topical application of 80% TCA solution.

## Introduction

Seborrheic keratoses (SKs) are common acquired benign epithelial tumor of the skin [1]. It is also known as seborrheic warts, senile warts, verrucosa seborrheic, and benign acanthokeratoma [2]. They can affect anywhere on the skin, except the palms and soles. SKs more commonly present as multiple, pigmented, with sharply demarcated lesions which begin as circumscribed brown patches or thin plaques. Over time, they may become papular or verrucous with greasy scale and stuck-on appearance [1]. A history of rapid growth, development of symptoms, color change, atypical morphology, or unusual location should prompt strong consideration for tissue biopsy and histologic examination [1]. Several studies have reported atypical cases such as auricular SKs, perianal giant SK, and nodular ulcerated achromic SKs [3]. Dermoscopy is a non-invasive examination tool that is gradually becoming widely used to diagnose atypical SKs [4]. Common standard treatments for SKs include curettage, shave excision, cryotherapy, electrocauterization, and laser therapy. In the past, topical application of chemicals, such as salicylic, glycolic, trichloroacetic (TCA), or pyruvic acid, either alone or in combination with surgical methods, has also been attempted [5]. We report a rare case of nodular SKs on the scalp which was diagnosed with dermoscopy and confirmed by histopathologic examination. The patient was successfully treated with topical application of 80% TCA solution.

## Patient and observation

**Figure 1:**
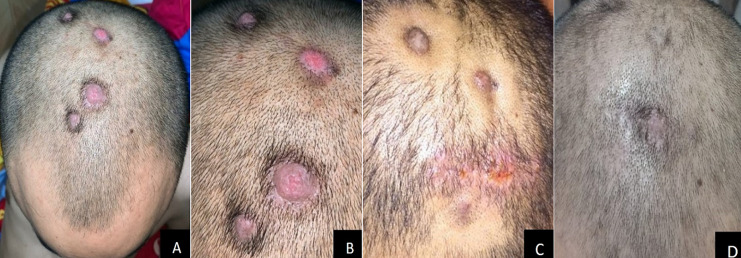
multiple well-circumscribed erythematous nodular hyperkeratosis on scalp (A, B) scar post excision appear closed and the other lesion showed thinning with minimal hyperpigmentation plaque. Four months post-treatment, further significant improvement of the lesions was evident, leaving only minimal hyperpigmentation macules without alopecia (C, D)

**Figure 2:**
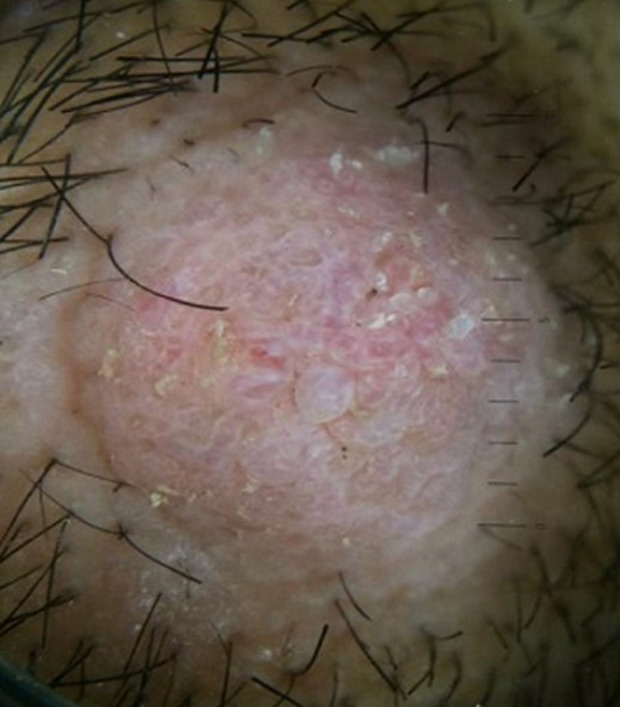
dermoscopic examination showed sharp demarcation, hairpin vessel and milia-like cyst

**Figure 3:**
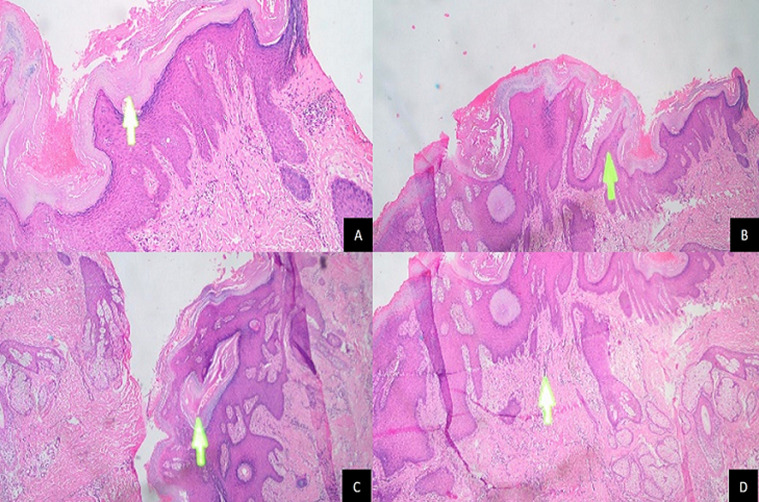
epidermal hyperplasia with hyperkeratosis (A), thick parakeratosis, hypergranulosis, papillomatosis (B) intraepidermal horn cyst (C) and dense inflammatory cell lymphocytes in the upper dermis (D)

A 39-year-old male was admitted to our clinic with multiple pruritic masses on the scalp that had been present for four months prior to admission. He had no known history of trauma. He also had no familial history of such lesion. Upon physical examination, multiple, well-circumscribed erythematous hyperkeratotic nodules with dome-shaped appearance were present on the scalp, with measured about 1-3cm in diameter. On palpation, the mass was firm in consistency with no tenderness nor discharge (Figure 1 A, B). Dermoscopic examination showed sharply demarcated border, hyperkeratosis, papillomatosis, hairpin vessel and milia like cyst (Figure 2 A) with no signs of malignancy. To confirm this finding, we conducted an excisional biopsy in the largest lesion to rule out any possibilities of malignancy under local anesthesia. Histological examination showed epidermal hyperplasia with hyperkeratosis, thick parakeratosis, hypergranulosis, papillomatosis, and an intraepidermal horn cyst. The upper dermis showed dense inflammatory lymphocyte (Figure 3). Based on the clinical, dermoscopic and histopathological findings, a diagnosis of SK was made. We decided to treat the remaining lesions with topical 80% TCA. Prior to TCA application, Vaseline was applied to the area surrounding the lesion to prevent irritation. The lesions were then cleansed with 70% ethanol before applying 80% TCA solution using a wooden applicator until a white frosting reaction was observed. Follow-up visit one week afterwards showed significant thinning of the lesions and hyperpigmentation plaque (Figure 1 C). The biopsied wound was well closed. Four months post treatment, further significant improvement of the lesions was evident, leaving only minimal hyperpigmentation macules without alopecia (Figure 1 D).

## Discussion

SKs are the most commonly reported benign skin tumor in elderly, usually above 50 years but young adult are also affected without any sexual predilection. Sun exposure has been postulated to play a central role in the development of SKs. Although the head is frequently exposed to sun light, the presence of hairs is thought to play a protective role [1]. Although rarely reported, SKs manifesting as nodules have been reported in multiple clinical presentations such as solitary ulcerated brown, well-demarcated, translucent nodule on chest and solitary brownish black nodule on the lower lid [6]. However, different to the aforementioned reports which showed solitary nodule of typical SK hyperpigmented appearance with relatively small sizes, the patient in our case presented with multiple hyperkeratotic erythematous nodules with “dome shaped” appearance on the scalp. This clinical presentation is very atypical for SKs and, to our knowledge, has never been reported. It is important to evaluate the possibility of malignancy when a lesion suspicious of SK is detected. In our patient, clinical differential diagnosis of mass was squamous cell carcinoma (SCC), dermatofibroma, and clear cell acanthoma. SCC mainly develop on sun-exposed sites and may metastasize. Its typical dermoscopic findings include bottom-hole vessels, white structureless areas and dotted or glomerular vessels [7]. Dermatofibromas present as firm, smooth papules or nodules. They frequently present on the extremities and trunk, asymptomatic and the lesions tend to be unchanged. Its classical dermoscopic appearance is white area resembling a scar surrounded by a discrete pigment network [8]. Clear cell acanthoma are a solitary, shiny, erythematous to brown, papule or nodule that almost completely pale with pressure and surrounding collarette of scale, dermatoscopic features are similar to psoriasis, with prominent vascular puncta [1]. The dermoscopic findings of SKs are presented according to Kittler *et al*. [4] where comedo-like openings, milia-like cysts, hairpin vessels and fissures and ridges are found to be characteristic of SKs. The correlation of clinical finding, dermoscopic and histology, confirmed the diagnosis of SK in our case.

The common operative methods for SKs include surgical resection, cryotherapy, electrodesiccation, or laser ablation. Complications of these methods include scarring, pigmentary alteration, incomplete removal, or even recurrence [1]. However, in case of extensive and/or multiple lesions, such as in this case, invasive procedures are often uncomfortable, impractical, and may require several sessions. In such cases, alternative treatments with topical application of chemical agents such as salicylic, glycolic, TCA, or pyruvic acid, either as monotherapy or in combination with surgical methods may be considered [5]. Higher TCA concentrations (>50%) should be used with caution due to the risk of post-inflammatory hyperpigmentation and scarring. The higher the concentration or amount of TCA applied, the more intense the destructive effect is. Therefore, it may represent an alternative strategy for skin tumor [9,10]. Considering the location, number and size of the lesion, we chose 80% TCA as the treatment option in this case. Effectiveness is seen only within one week after treatment, where the lesions showed a significant reduction in size and thickness, leaving minimally hyperpigmented plaques. At the fourth month of follow-up, the lesion almost completely resolved. In addition, hair growth was observed on the treated area, showing that scarring did not take place. This showed that TCA is effective in SK treatment and can be considered as an alternative therapy in cases with large and/or multiple lesions. A similar result was reported by Sea *et al*. who showed that focal application of 100% TCA showed a significant clinical improvement in patients with scalp SKs [10].

## Conclusion

Seborrheic keratosis on the scalp is a rare finding and reports are still limited. Although the risk of progression to malignancy is low, they often tend to increase in size and number with advancing age, sometimes raising cosmetic concerns. Focal application of 80% TCA was effective and is a safe noninvasive choice of therapy in the treatment of scalp SKs.

## References

[1] Jonathan DC, Sewon k (1799). Seborrheic keratosis on benign epithelial tumor, hamartomas and hyperplasias. fitzpatricks dermatology 2019.

[2] Thappa DM, Malathi M (2017). Seborrheic keratosis.

[3] Hong CE, Kim Y, Choi HS, Jeong J (2019). Seborrheic Keratosis in the Auricle: a rare tumor. Ear Nose Throat J.

[4] Kittler H, Marghoob AA, Argenziano G, Carrera C, Curiel-Lewandrowski C, Hofmann-Wellenhof R (2016). Standardization of terminology in dermoscopy/dermatoscopy: results of the third consensus conference of the International Society of Dermoscopy. J Am Acad Dermatol.

[5] Lacarrubba F, Nasca MR, Verzi AE, Micali G (2017). A novel topical agent in the treatment of seborrheic keratoses: a proof of concept study by clinical and dermoscopic evaluation. Dermatol Ther.

[6] Mishra DK, Jakati S, Dave TV, Agrawal M (2019). A rare pigmented lesion of the eyelid. Int J Trichology.

[7] Gaballah MA, Ahmed RA (2015). Diagnostic value of CD10 and Bcl2 expression in distinguishing cutaneous basal cell carcinoma from squamous cell carcinoma and seborrheic keratosis. Pathol Res Pract.

[8] Barroso DH, Leite CPZ, Araujo GDdS, Teixeira MAG, Alencar ERB, Cavalcanti SMdM (2016). Dermatofibroma simulating seborrheic keratosis dermoscopically. An Bras Dermatol.

[9] Harmon CB, Hadley M, Tristani P (2011). Trichloroacetic acid. Color atlas of chemical peels: springer.

[10] Seo HM, Lim JY, Choi JY, Kim HS, Kim JI, Min J (2015). P587: focal application of 100% trichloroacetic acid for the treatm+ent of seborrheic keratosis on the scalp area: a case series.

